# The Impact of Varying Cooling and Thawing Rates on the Quality of Cryopreserved Human Peripheral Blood T Cells

**DOI:** 10.1038/s41598-019-39957-x

**Published:** 2019-03-04

**Authors:** Jasmin Baboo, Peter Kilbride, Mike Delahaye, Stuart Milne, Fernanda Fonseca, Magdalena Blanco, Julie Meneghel, Alex Nancekievill, Nick Gaddum, G. John Morris

**Affiliations:** 1 Cell and Gene Therapy Catapult, 12th Floor Tower Wing, Guy’s Hospital, Great Maze Pond, London, SE1 9RT UK; 2 Asymptote, General Electric Healthcare, Sovereign House, Histon, Cambridge, CB24 9BZ UK; 3 UMR GMPA, AgroParisTech, INRA, Université Paris Saclay, 78850 Thiverval-Grignon, France

## Abstract

For the clinical delivery of immunotherapies it is anticipated that cells will be cryopreserved and shipped to the patient where they will be thawed and administered. An established view in cellular cryopreservation is that following freezing, cells must be warmed rapidly (≤5 minutes) in order to maintain high viability. In this study we examine the interaction between the rate of cooling and rate of warming on the viability, and function of T cells formulated in a conventional DMSO based cryoprotectant and processed in conventional cryovials. The data obtained show that provided the cooling rate is −1 °C min^−1^ or slower, there is effectively no impact of warming rate on viable cell number within the range of warming rates examined (1.6 °C min^−1^ to 113 °C min^−1^). It is only following a rapid rate of cooling (−10 °C min^−1^) that a reduction in viable cell number is observed following slow rates of warming (1.6 °C min^−1^ and 6.2 °C min^−1^), but not rapid rates of warming (113 °C min^−1^ and 45 °C min^−1^). Cryomicroscopy studies revealed that this loss of viability is correlated with changes in the ice crystal structure during warming. At high cooling rates (−10 °C min^−1^) the ice structure appeared highly amorphous, and when subsequently thawed at slow rates (6.2 °C min^−1^ and below) ice recrystallization was observed during thaw suggesting mechanical disruption of the frozen cells. This data provides a fascinating insight into the crystal structure dependent behaviour during phase change of frozen cell therapies and its effect on live cell suspensions. Furthermore, it provides an operating envelope for the cryopreservation of T cells as an emerging industry defines formulation volumes and cryocontainers for immunotherapy products.

## Introduction

The demand for robust and effective T cell therapies has grown rapidly based on their potential to treat many forms of cancer^1–3^. With the recent advancements and the first ever chimeric antigen receptor T cell (CAR T) therapy, Yescarta^TM^ being approved by the Food and Drug Administration (FDA) in 2017^4^, implementation of a cryochain will be essential both for effective clinical delivery and to provide a commercially robust business model. Just in Time (JIT) manufacture or short-term storage at 4 °C may be appropriate to some early stage clinical trials, however successful cryopreservation allows efficiencies in manufacturing, facilitates quality control, enables flexibility in patient scheduling and allows transport of the therapy to the bedside.

There are several studies on the cryopreservation of peripheral blood mononuclear cells (PBMCs) and purified T cells examining their sensitivities to cell density^5,6^, cryoprotectant additives^7,8^, a variety of cooling rates^9–13^ fluctuations in storage temperatures^14^ and period of liquid nitrogen storage^15^. Furthermore, Quality-by-Design (QbD) approaches have been used as a predictive tool for optimizing human embryonic stem cell (hESC) cryopreservation and revival processes^16^. By contrast the effects of warming rates have received very little attention. One study^12^ showed that warming rates in the range 100 °C min^−1^ to 2 °C min^−1^ had very little effect on the viability of mouse lymphocytes following a single slow rate of cooling with DMSO as cryoprotectant; only at very slow rates of warming was viability compromised. We know of no systematic study where the effects of thawing rate on the outcomes of cryopreservation of T cells has been published. It is commonly assumed that cryopreserved cells must be thawed rapidly otherwise ice recrystallization events occur during warming which are detrimental to cell recovery^17–21^. Whilst this may be true for spermatozoa which have been cooled rapidly with glycerol as a cryoprotectant^22,23^, the evidence for somatic mammalian cells which have been cooled at slow rates with DMSO as cryoprotectant is lacking^24,25^.

For the clinical delivery of T cell therapies, it is critical to understand the cryomechanics of cooling and warming cell material in order to ensure reproducibility, maximise cost per dose, viability and cell function before delivery to the patient. Rapid thawing protocols typically involve placing the samples into a 37 °C water bath. This is problematic as water baths are not compatible with current good manufacturing practice (cGMP) requirements and should not be used in operating theatres or cleanrooms^26–28^. Therefore, alternative warming strategies must be employed in a clinical setting, but these would be expected to result in less rapid heat transfer into the sample. Additionally, because of the need to maintain sterility of parenterally delivered treatments classical screwcap cryovials will likely be replaced with newer hermetically sealed vials which employ plastics with lower thermal conductivity and higher capacity, or with cryobags of large volumes (typically 50 mL to 250 mL). This also would be expected to influence the rate of thawing of the samples.

In this study, we examine the interaction between the rate of cooling and rate of warming on the viability and function of T cells formulated in a conventional DMSO based cryoprotectant and processed in conventional cryovials. In parallel, a cryomicroscopy study allowed the ice structure formation during both cooling and thawing to be observed which allowed phenomenon measured in the viability and functional study to be explained. Observations in the cryomicroscopy study could be quantified using differential scanning calorimetry (DSC) which measures ice formation through observed heat flow. Finally, an estimation is presented to allow scaling from the conventional cryovial format used in this study. In this case, predictions are made as to whether the rate of thawing would become limiting to the efficacy of the clinical treatment when using new generation vials of lower conductivity/larger capacity and bags of larger volumes.

We anticipate that this study would also be relevant to the cryopreservation of the initial leukapheresis sample which is the source of the derived T cells for autologous immunotherapies, and to a broader range of cells and gene therapies.

## Materials

### Reagents

The leukapheresis pack (#PB001F-1) was obtained fresh from Hemacare (Los Angeles, CA, United States). The GMP grade Dynabeads® human T-Expander CD3/CD28 (#11141D), carboxyfluorescein succinimidyl ester (CFSE) (#C1157), non-GMP Dynabeads® human T activator CD3/CD28 (#11131D), Dulbecco’s phosphate buffer saline (-Mg, -Ca) (PBS) (#14190250), live/dead fixable aqua dead cell stain (live/dead aqua) (#L34957) and 16% formaldehyde (#28908) were obtained from ThermoFisher Scientific (Waltham, MA, United States). The CryoStor10® (#C2874) was obtained from Sigma-Aldrich (St. Louis, MO, United States). The XVIVO 15 without gentamycin and phenol red (XVIVO 15) (#LZBE02-061Q) was obtained from SLS (Hessle, Yorkshire). The human AB serum (#BRH1053237) was obtained from Seralab (West Sussex, UK). The pro-leukin (IL-2) (#pro-leukin) was obtained from Novartis (Basel, Switzerland). The anti-human CD3 PE Vio770 (#130-096-749), anti-human CD4 PE (#130-091-231) and anti-human CD8 APC Vio770 (#130-091-561) were obtained from Miltenyi Biotec (Cologne, Germany). The DMSO (#276855) was obtained from Sigma-Aldrich (St. Louis, MO, United States). The staining buffer (#554656) was obtained from Becton Dickson (Oxford, UK).

### Cell Line

T cells were originally expanded from a fresh leukapheresis pack by stimulation with GMP grade Dynabeads® human T-Expander CD3/CD28 and frozen (−1 °C min^−1^ to −80 °C followed by long term storage in LN_2_) in 1 mL aliquots using 2 mL cryovials (Corning, sourced from Sigma-Aldrich #CLS430489, St. Louis, MO, United States) at 1 × 10^7^ cells mL^−1^ formulated in CryoStor10.

## Methods

**Figure 1:**
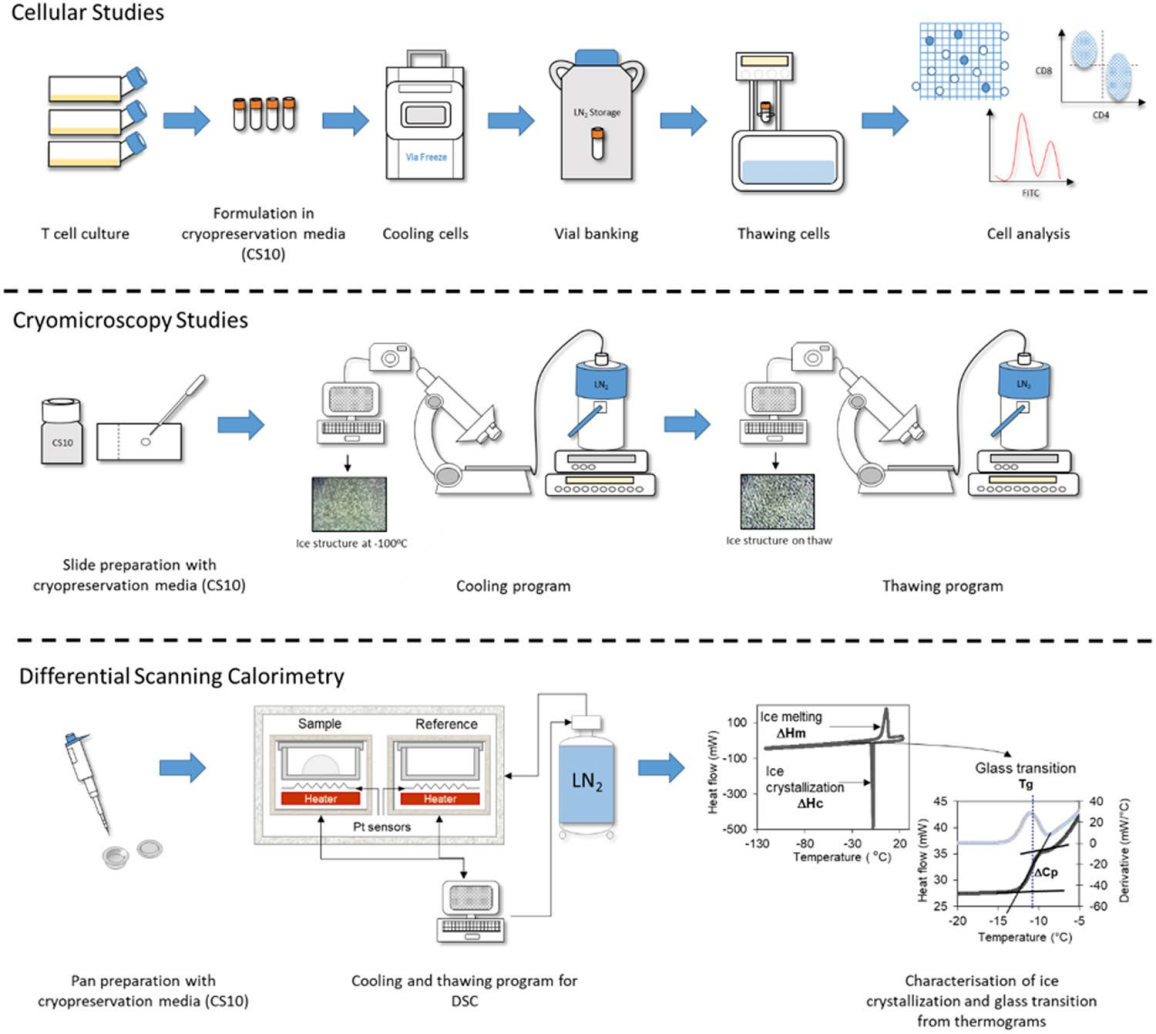
Schematic representation of the cellular studies, cryomicroscopy studies and differential scanning calorimetry conducted. Full details of the methods followed are described in the methods section. For cellular studies T cells were cultured for seven days in T175 flasks and then formulated in CryoStor10 cryopreservation media at 1 × 10^7^ cells mL^−1^. The cells were frozen down using four cooling rates 0.1 °C min^−1^ (using a VIA freeze controlled rate freezer), 1 °C min^−1^, 10 °C min^−1^ (using a Planer controlled rate freezer) and ~159 °C min^−1^ (immersed in LN_2_) and then thawed at four thawing rates: a very slow thaw (in a polystyrene insert), a slow thaw (in air), a standard thaw (in a 37 °C water bath) and a rapid thaw (in a 95 °C water bath). The thawed cells were then analysed using viability, phenotype and proliferation assays to determine the impact on cellular performance. For cryomicroscopy studies CryoStor10 cryopreservation media was added to a microscope slide and this was placed on a microscope with an attached BC196 cold stage system using LN_2_ cooling. Cooling and thawing protocols were programmed into the cold stage system and by using a camera attached to the optical output of the microscope videos of the whole cooling-thawing process were captured on a laptop and images taken at appropriate time points. A DSC protocol was used for determining the ice crystallization and glass transition temperature of cryopreservation media (CryoStor10) at different cooling and warming rates.

The various methods used in this study are summarised in Fig. 1.

### Cell culture

Four T175 flasks (Nunc, sourced from VWR, #7342129, Radnor, PA, United States) were seeded with 1 × 10^6^ T cells mL^−1^ in 100 mL XVIVO 15 + 5% AB serum, 300 units mL^−1^ IL-2 and 1 × 10^6^ GMP grade CTS Dynabeads CD3/CD28 mL^−1^. The flasks were incubated at 37 °C, 5% CO_2_ and 21% O_2_ for two days in a NuAire incubator (NuAire, Plymouth, MN, United States). Samples of the seeded cells were taken and stained with CD3, CD4 and CD8 antibodies to assess the cell phenotype as described in the phenotype analysis section, stained with CFSE and seeded for a proliferation assay as described in the proliferation analysis section. The flasks were split 1:1 on day two (into 8 flasks) and four (into 16 flasks) where 50 mL of cell suspension was added to 50 mL of fresh XVIVO 15 + 5% AB serum (containing 300 units mL^−1^ of IL-2) and then returned to the incubator at 37 °C, 5% CO_2_ and 21% O_2_. On day seven samples were taken from the flasks and counted using a Vicell XR automated cell counter (Beckman Coulter, High Wycombe, UK) which uses Trypan blue staining and the required number of cells were harvested from the flasks by centrifuging the cell suspension at 1000 g for 15 minutes in an Allegra X-15R centrifuge (Beckman Coulter, High Wycombe, UK), the supernatant was discarded and the cell pellet resuspended in XVIVO 15 + 5% AB serum. The resuspended cells were placed in a DynaMag-50 magnet (ThermoFisher Scientific, Waltham, MA, United States) for two rounds of 3 minute separations to remove the GMP grade CTS Dynabeads CD3/CD28. Samples were taken from the cell suspension for a cell count using the Vicell, for phenotype analysis and for proliferation analysis. Cells were then prepared for cryopreservation.

### Cryopreservation protocol

The required cells were centrifuged at 1000 g for 15 minutes and the supernatant discarded. The cell pellet was resuspended in CryoStor10 chilled to 4 °C to achieve a cell concentration of 1 × 10^7^ cells mL^−1^ and 1 mL aliquots dispensed into 2 mL cryovials (Corning, sourced from Sigma-Aldrich #CLS430489, St. Louis, MO, United States). Vials were immediately cooled using three linear cooling rates, 0.1 °C min^−1^ from 4 °C to −100 °C using a VIA Freeze controlled rate freezer (Asymptote, GEHC, Cambridge, United Kingdom), 1 °C min^−1^ and 10 °C min^−1^ down to −100 °C using a Planer Kryo 550 controlled rate freezer (Planer, Middlesex, UK), and immersed in liquid nitrogen (LN_2_) for a period of 2 minutes (~159 °C min^−1^). Once cooling was complete all vials were transferred directly to LN_2_ until required for warming studies; ice nucleation was not induced.

### Warming process

Vials were removed from LN_2_ and placed into the VIA Freeze controlled rate freezer (Asymptote, GEHC, Cambridge, United Kingdom) at −100 °C for a minimum of 30 minutes. This 30 minute hold at −100 °C ensured a consistent starting point for all conditions tested. The vials were then warmed using four methods to achieve pre-determined thermal profiles:

Water bath thawing – a temperature controlled water bath (Grant JB Academy, Cambridge, United Kingdom) was set and equilibrated to the desired temperature (37 °C or 95 °C). Above this, a custom-made robotic cryovial holder was attached. This was then lowered into the bath using an automatic pre-defined program (for 2 minutes 50 seconds at 37 °C and for 1 minute 20 seconds at 95 °C) to ensure consistency between warms.

Warming in air – samples were removed from the freezer and placed upright on the bench until warmed (16 minutes and 30 seconds). Samples were not touched or in any way agitated during this process.

Warming in polystyrene – a polystyrene insert was constructed consisting of a large volume of polystyrene with inserts for cryovials. Samples were removed from the freezer and added to the polystyrene until warmed (53 minutes).

### Determination of thermal profiles

Cryovials (2 mL, Corning, sourced from Sigma-Aldrich #CLS430489, St. Louis, MO, United States) were filled with 1 mL CryoStor10 cryoprotectant (#C2874, Sigma-Aldrich). A K-type thermocouple was added to each of at least 5 vials. These thermocouples were attached to a picologger unit (Picotechnology, St. Neots, United Kingdom), and temperatures recorded using picologger software (Picotechnology, St. Neots, United Kingdom).

### Cell counts

Once the vials had been thawed, the vial content was mixed and the cells added to a 15 mL falcon tube (VWR, #7340448, Radnor, PA, United States). 4 mL of XVIVO 15 + 5% AB serum was added dropwise to the cells and then slowly pipetted up and down to re-suspend cells. The cells were centrifuged at 500 g for 5 minutes at room temperature (maximum acceleration and deceleration used throughout unless otherwise stated) and the supernatant discarded. The cell pellet was resuspended in 10 mL of XVIVO 15 + 5% AB serum using a 10 mL stripette and the cells counted using the Vicell which measures both the total and viable cells in the sample.

### Phenotype analysis

The phenotype of the cell populations were analysed by staining with three standard T cell surface markers CD3, CD4 (T helper cells) and CD8 (T killer cells). The cells were prepared according to the following procedure.

Once cells had been counted a sample 1 × 10^6^ viable cells was retrieved and washed with an equal volume of staining buffer. The sample was centrifuged for 5 minutes at 500 g at room temperature and the supernatant discarded, with the pellet resuspended in 700 µL of PBS. 100 µL of this sample was left unstained and added to a 96-well plate U-bottom shape (SLS, #351177, Hessle, Yorkshire) while the remaining 600 µL was stained with 1 µL of live/dead aqua stain and incubated for 30 minutes in darkness. Once incubation was complete the cells were washed with 500 µL of PBS and mixed. The sample was centrifuged for 5 minutes at 500 g at room temperature and the supernatant discarded, with the pellet resuspended in 600 µL of staining buffer. The sample was then split into six, and cells were incubated with the antibodies CD3, CD4 and CD8 in a 96-well plate U-bottom shape. Three of the samples were fully stained with all three antibodies and the last three samples where incubated with antibodies to be used as fluorescence minus one (FMO) controls (FMO CD3 – incubated with CD4 and CD8, FMO CD4 – incubated with CD3 and CD8, FMO CD8 – incubated with CD4 and CD3). The samples were incubated for 20 minutes in the fridge. Then, the plate was centrifuged for five minutes at 500 g at room temperature and the supernatant discarded, with the pellets resuspended in 200 µL of staining buffer. The cells were subsequently fixed using 4% formaldehyde, stored at 2–8 °C and analysed on the MACSQuant analyser 10 flow cytometer (Miltenyi Biotec, Cologne, Germany) after 24 hours.

### Proliferation analysis

The level of proliferation was analysed by using a CFSE proliferation assay. Cells were first labelled with CFSE which gives a green fluorescence and cell division was measured by the halving of the fluorescence intensity of CFSE after a 4 day incubation. Cells were prepared for the assay according to the following procedure.

Once cells had been counted a sample of 6 × 10^6^ total cells was mixed with 10 mL of PBS and centrifuged for 5 minutes at 500 g at room temperature. The supernatant was discarded and the pellets resuspended in 1 mL of 2.5 μM CFSE in DMSO. The cells were then incubated for 5 minutes in the dark at room temperature and then mixed with 10 mL of XVIVO 15 + 5% AB serum. The cells were centrifuged for 5 minutes at 500 g at room temperature and the supernatant discarded, with the pellets resuspended in 1 mL of XVIVO 15 + 5% AB serum and a sample taken for a cell count using the Vicell. After the cell count, cells were resuspended in XVIVO 15 + 5% AB serum to achieve a cell density of 1 × 10^6^ total cells mL^−1^. Cells were subsequently seeded (1 × 10^5^ cells per well) in XVIVO 15 + 5% AB serum (unstimulated) or with non-GMP CTS Dynabeads CD3/CD28 at a 1:1 (1 × 10^5^ beads: 1 × 10^5^ cells) ratio (stimulated) in a 96-well plate U-bottom shape. The plate was incubated at 37 °C, 5% CO_2_ for 4 days and then centrifuged for 5 minutes at 500 g at room temperature. The supernatant was discarded and the pellets were resuspended in 200 µL PBS. Cells were analysed using the MACSQuant analyser 10 flow cytometer. The level of proliferation was measured from the reduction in CFSE fluorescence in stimulated cells compared to unstimulated cells and was expressed as a percentage of proliferating cells in the whole population.

### Cryomicroscopy

Cryomicroscopy images were taken on an Olympus BX51 microscope (Tokyo, Japan) with an attached BCS196 cold stage system (Linkam Scientific, Tadworth, United Kingdom) using liquid nitrogen cooling to control temperature. A camera was attached to the optical output of the microscope.

CryoStor10 was added to a microscope plate, with a quantity of IceStart (Asymptote, GEHC, Cambridge, UK) included to prevent excessive supercooling of the sample. The desired cooling and warming program was entered into the Linkam cold stage system before the cooling and thawing protocol commenced. The camera was connected to a laptop and recorded a video of the whole cooling-warming process. Images were taken from this video at the appropriate time points. The thawing profiles used on the cryomicroscope emulate the measured temperature profiles that actually occur in vials determined by thermocouples and shown in Fig. 1.

### Differential scanning calorimetry (DSC)

DSC allows the characterization of different thermal events taking place in a complex liquid sample such as CryoStor10 during freeze-thawing (e.g. ice crystallization, glass transition, ice melting). DSC measurements were carried out using a power compensation DSC (Diamond, Perkin Elmer LLC, Norwalk, CT, USA) equipped with a liquid nitrogen cooling accessory (CryoFill, Perkin Elmer). Temperature calibration was performed using a known mass of cyclohexane (crystal-crystal transition at −87.1 °C), mercury and gallium (melting points at −38.6 °C and +29.8 °C, respectively). Latent heat (delta-H) calibration was performed with mercury (melting enthalpy, ΔHm, 11.6 J g^−1^). About 5 mg of CryoStor10 were placed in 50 µl Perkin Elmer DSC sealed aluminium pans. An empty pan was used as a reference. Different linear cooling and warming rates (only the curves obtained during warming are displayed as glass transition is observed during warming by DSC) were applied throughout these DSC studies: 2, 5, 10, 100 and 150 °C min^−1^ for cooling, and 2, 5 and 10 °C min^−1^ for warming. Samples were scanned during cooling to −150 °C and then during warming to 25 °C. The extent of ice crystallization was characterised by the latent heat released during this thermal event (ΔHc, J g^−1^), which was quantified by the area under the associated exothermic peak. Similarly, the extent of ice melting was characterised by the absorbed latent heat (ΔHm, J g^−1^) and quantified by the area under the associated endothermic peak. Different values of latent heat between ice crystallization and melting indicate a deviation from the equilibrium freezing behaviour, therefore the occurrence of recrystallization phenomena.

Glass transition of biological medium refers to the transition from a viscoelastic to a glassy state during cooling and vice versa during warming and it is associated with strong modifications of the physical properties of the samples (viscosity, molecular mobility). The glass transition of CryoStor10 samples were observed by DSC during warming. Glass transition is usually characterized by two consecutive thermal events: Tg_1_ and Tg_2_. The lower value (Tg_1_) represents the glass transition temperature of the freeze concentrated phase^29,30^ and the higher value (Tg_2_) represents the softening temperature at which the system exhibits an observable deformation (viscous flow in real time) under its own weight^31^. Tg_1_ and Tg_2_ of CryoStor10 samples were determined as the midpoint temperatures of the heat flow steps associated to the glass transition with respect to the ASTM Standard Method E 1356-91. Results were obtained from at least four replicates.

### Heat transfer calculations

To determine approximate rates of warming in cryocontainers other than the 2 ml cryovials in this study, several vials and bags were acquired with their dimensions and wall thicknesses measured using callipers (RS Components, 146–5498, Corby, UK). Using known heat transfer coefficients for EVA (Ethylene vinyl acetate) and COC (Cyclic olefin copolymer) between 0.1 and 0.2 W/(m.k), the rate of heat transfer relative to that of a cryovial was calculated per unit volume of solution.

### Statistical analysis

Statistical analyses of Tg_1_, Tg_2_, ΔHm, ΔHc and ΔHm + ΔHc of CryoStor10 according to different cooling and warming rates from at least four replicates (DSC data) were performed in R 3.4.2 using the R Commander package. After testing for normality of distributions (Shapiro-Wilk test) and for homogeneity of variances (Bartlett and Levene’s tests) at a 95% confidence interval, statistical comparisons were made with one-way ANOVA through pairwise comparisons of means.

## Results

### Measured warming rates

**Figure 2:**
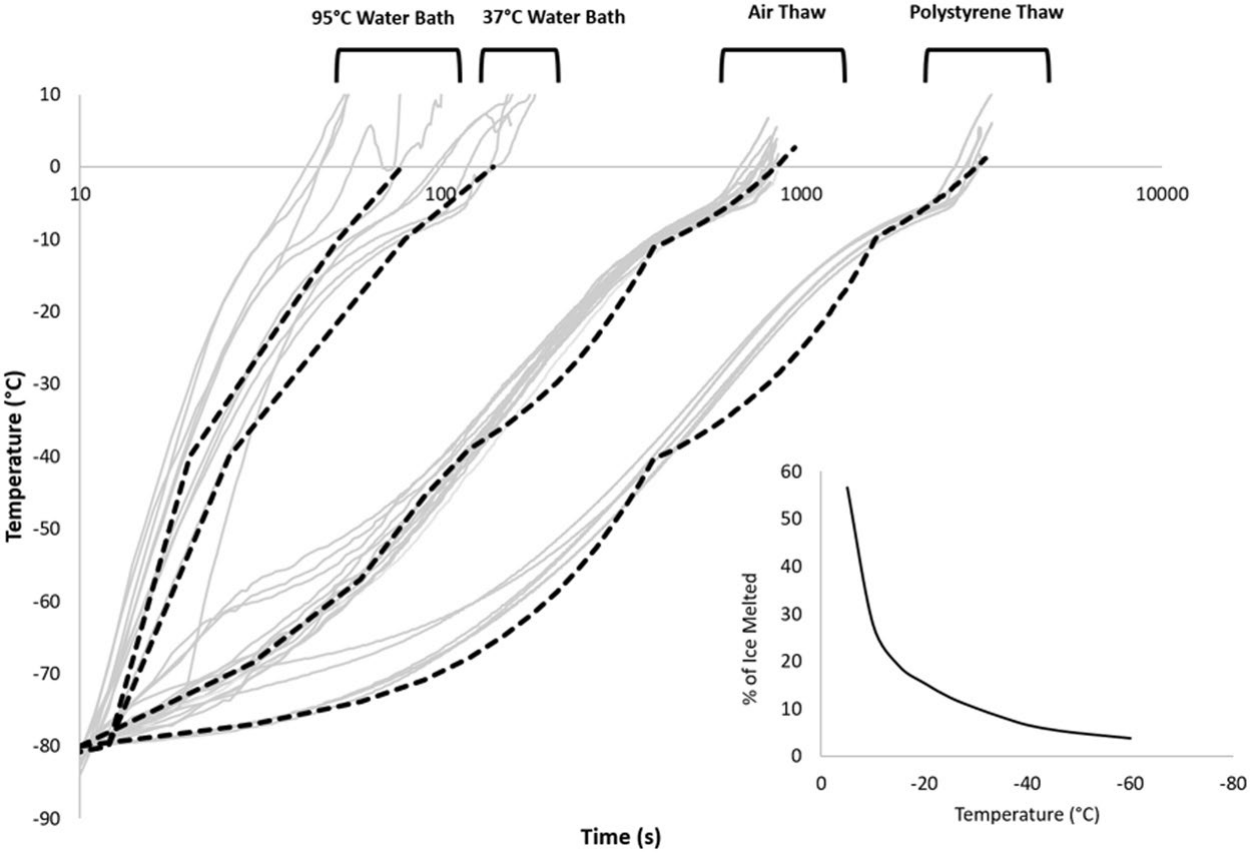
Measured temperatures in cryovials (1 mL fill) during various warming protocols (grey solid line). The warming rates between −80 °C and 0 °C were 113 ± 37 °C min^−1^ and 45 ± 8 °C min^−1^ in the 95 °C and 37 °C water baths, respectively, 6.2 ± 0.5 °C min^−1^ for samples thawed in air and 1.6 ± 0.1 °C min^−1^ in samples thawed in polystyrene. These profiles were emulated using the cryomicroscope (black dashed line) in order to visualize any changes in ice structure during warming in vials. The insert graph shows the total mass of a 10% DMSO system in the liquid state at any given temperature, derived from^43^.

The measured rates of warming achieved in vials by the different experimental protocols are shown in Fig. 2. The warming rates measured between −80 °C and 0 °C range from an average of 113 ± 37 °C min^−1^ and 45 ± 8 °C min^−1^ in the 95 °C and 37 °C water baths, respectively, to 6.2 ± 0.5 °C min^−1^ for samples thawed in air and 1.6 ± 0.1 °C min^−1^ in samples thawed in polystyrene. In all cases the rates of warming are observed to be nonlinear with time. Samples thawed in the 95 °C water bath warmed at an average rate of 293 ± 29 °C min^−1^ between −80 °C and −40 °C, but only 39 ± 23 °C min^−1^ between −10 °C and −1 °C. This compares with an average rate of 6.4 ± 0.4 °C min^−1^ between −80 °C and −40 °C, and 0.41 ± 0.03 °C min^−1^ between −10 °C and −1 °C when the polystyrene insert was used.

### Ice structure and quantity

**Figure 3:**
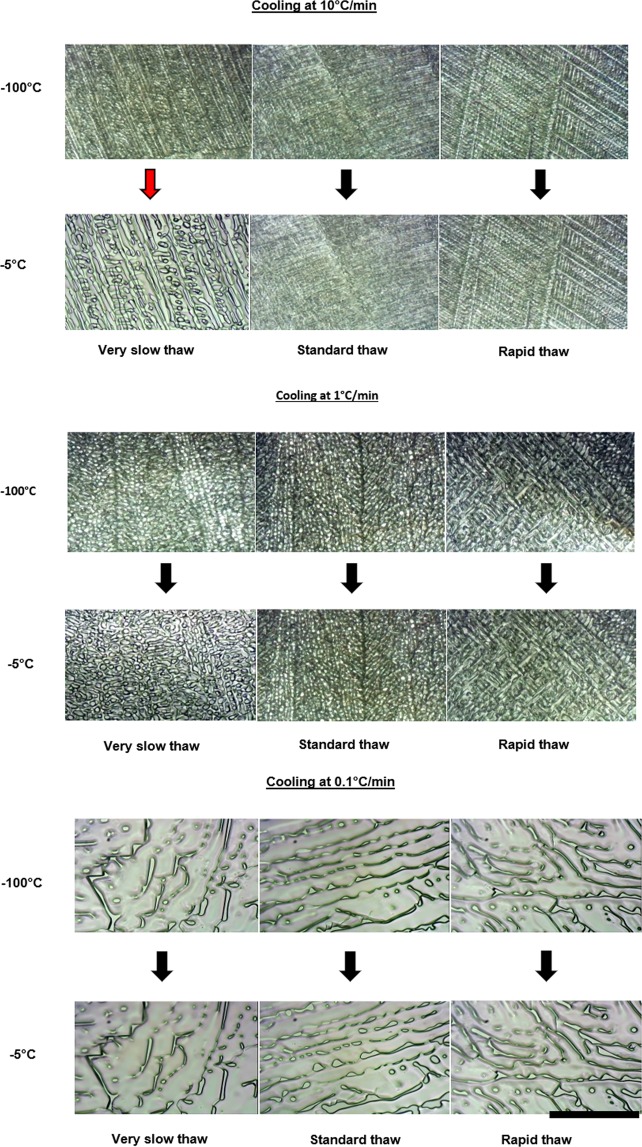
Ice structure and changes observed during the warming cycle. Each of the three sets show different cooling rates (10 °C min^−1^, 1 °C min^−1^, and 0.1 °C min^−1^ from top to bottom respectively). The upper three images in each set show the ice structure on reaching −100 °C, and the lower three images show the ice structure just prior to melting (at approximately −5 °C). In each set three different warming profiles were carried out. From left to right very slow (in polystyrene), standard (in a 37 °C water bath), and rapid thaws (in a 95 °C water bath). Ice structure changes are shown by the red arrow. The scale bar indicates approximately 100 μm, with each image shown to the same scale.

A cryomicroscope was employed to examine the ice structure and changes in ice structure following different combinations of cooling and warming rates. The rates of warming used in the cryomicroscopy studies are the non-linear profiles measured within cryovials (Fig. 2). As can be seen in Fig. 3, for the standard cryopreservation protocol of cooling at 1 °C min^−1^ and thawing in a 37 °C water bath, changes in ice structure occur during the cooling cycle, but there is little apparent change on warming, until complete thawing of the sample occurs. This is also true of samples cooled at 10 °C min^−1^ and warmed in a water bath. Samples cooled at 10 °C min^−1^ but warmed very slowly in polystyrene exhibit substantial ice structure change during warming. Samples cooled at 0.1 °C min^−1^ have a much larger ice crystal structure after cooling, but the ice structure was not modified during warming under any warming condition. Videos of cryomicroscopy are included in Supplementary Information.

### Differential scanning calorimetry (DSC)

During cooling, ice crystallization of CryoStor10 was visualised by DSC as an exothermic event releasing heat, therefore resulting in a heat flow measured by DSC (ΔHc in Table 1) whereas ice melting occurring during warming appeared as expected as an endothermic event (ΔHm in Table 1). The difference between the heat flows associated to both events (ΔHm − ΔHc) informs about a potential deviation from equilibrium freezing behaviour of the sample. Positive values indicate a higher quantity of ice melting than has crystallized during cooling, suggesting recrystallization during warming. Here, cooling rates of 10 °C min^−1^ or below followed by 10 °C min^−1^ thawing resulted in ΔHm − ΔHc values close to zero (Table 1). When increasing the cooling rate to 100 and 150 °C min^−1^, a gradual increase of ΔHm − ΔHc was observed. The difference of heat flow reached about 10 J g^−1^ for cooling rates of 150 °C min^−1^ and thawing rates between 10 and 2 °C min^−1^ (with no significant differences between thawing rates, *p*-value = 0.98), This result indicates a significant deviation from equilibrium freezing behaviour and suggests that some water that failed to crystallize during fast cooling, could recrystallize during thawing.

**Table 1:** Summary of the thermal events observed by DSC of CryoStor10 samples following freezing and thawing at different cooling and warming rates: glass transition temperatures (Tg_1_) and softening temperature (Tg_2_), heat of crystallization (ΔHc), heat of melting (ΔHm). Superscript letters indicate statistical contrasts between means for the different cooling and warming rates applied at a 95% confidence level.

Cooling rate (°C min^−1^)	Warming rate (°C min^−1^)	Tg_1_ (°C)	Tg_2_ (°C)	ΔHc (J g^−1^)	ΔHm (J g^−1^)	ΔHm − ΔHc (J g^−1^)
2, 5	10	−121.0 ± 1.6^a^	−72.9 ± 3.4^a^	155.4 ± 3.6^ab^	157.4 ± 4.2^a^	2.1 ± 4.1^ab^
10	10	−121.3 ± 2.1^a^	−70.6 ± 1.8^ab^	156.9 ± 4.3^a^	157.4 ± 3.6^a^	0.5 ± 4.0^a^
100	10	−122.4 ± 2.4^a^	−68.1 ± 2.1^b^	153.1 ± 3.2^ab^	157.4 ± 3.2^a^	4.4 ± 2.6^ac^
150	10	−122.6 ± 1.2^a^	−68.1 ± 1.9^b^	149.9 ± 2.8^ab^	159.6 ± 3.6^a^	9.7 ± 5.1^bc^
150	2, 5	−122.7 ± 1.7^a^	−69.3 ± 1.8^ab^	148.8 ± 6.3^b^	160.0 ± 4.0^a^	11.1 ± 6.7^c^

**Figure 4:**
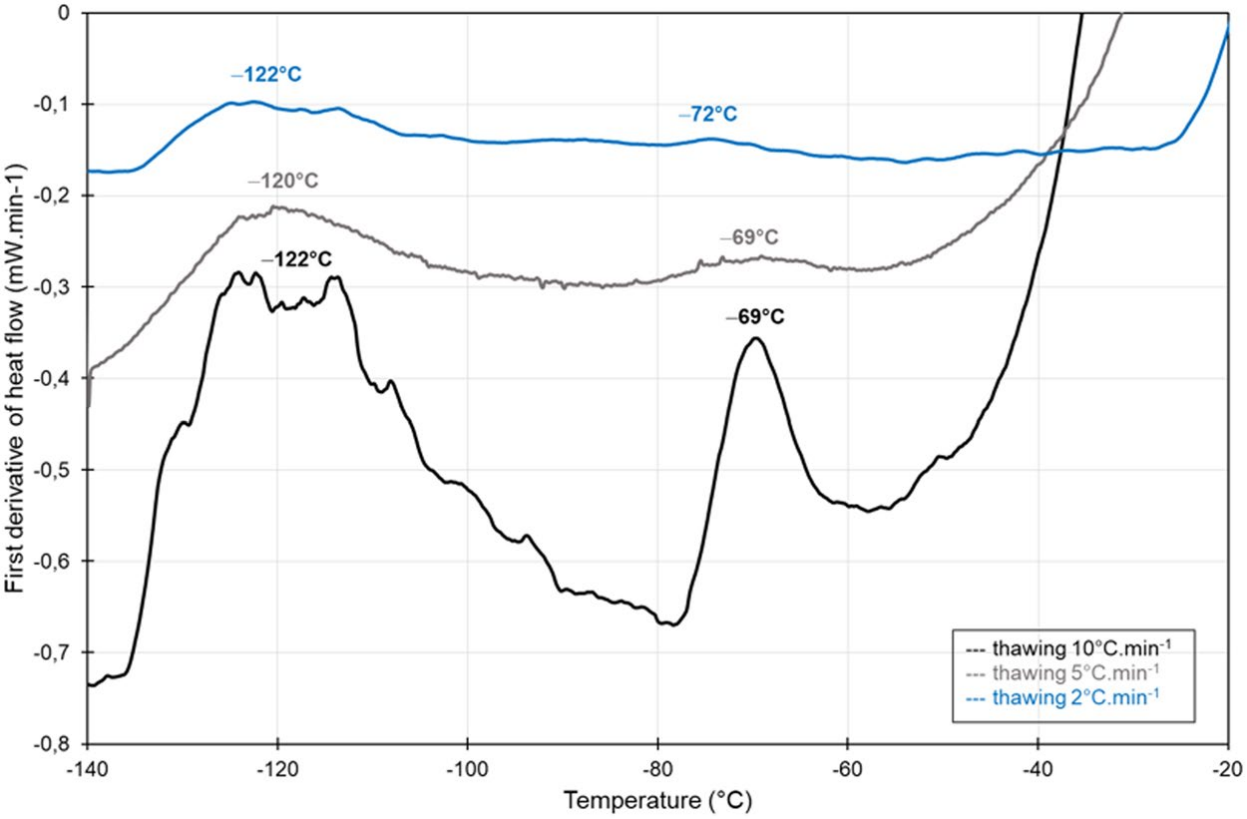
First derivatives of heat flow traces of CryoStor10 obtained by DSC following cooling at 150 °C min^−1^ and thawing at different rates from 2 to 10 °C min^−1^. Both endothermic events at approximately −120 °C and −70 °C represent the glass transition temperature (Tg_1_) and the softening temperature (Tg_2_), respectively.

Upon warming, two weak (explained by the complex composition and low concentrations) endothermic events were identified from the first derivative of the heat flow at about −120 °C (Tg_1_) and −70 °C (Tg_2_) (Table 1 and peaks in Fig. 4). They correspond to the glass transition temperature of the freeze concentrated phase and the softening temperature, respectively^32^. However, no exothermic peak corresponding to the ice recrystallization event was identified during thawing (Fig. 4). No significant influence of the cooling nor warming rates could be observed on Tg_1_ (*p*-value > 0.5, Table 1 and Fig. 4). Slight shifts of Tg_2_ values were observed when the cooling rate was increased or the thawing rate was decreased. However, the low Tg_2_ signals observed at low thawing rates limit any further interpretation (Fig. 4).

### Viability tests

Trypan blue viability assays shown in Supplementary Materials Fig. S2A show that samples cooled at 0.1 °C min^−1^ or 10 °C min^−1^ had significantly (*p*-values < 0.05 and <0.01, respectively) lower viability compared with 1 °C min^−1^ cooling when thawed in polystyrene. In addition, samples cooled at 0.1 °C min^−1^ had a significantly higher viability compared with 1 °C min^−1^ cooling when thawed in a 95 °C water bath (*p*-value < 0.05). No significant differences were seen with any other warming profile compared to the 1 °C min^−1^ cooling condition. Very poor (<40%) viability was seen in samples which were plunged into LN_2_ directly (159 °C min^−1^) therefore the data is not shown.

Using live/dead aqua staining for phenotype analysis Supplementary Fig. S2B shows samples cooled at 10 °C min^−1^ had significantly worse outcome when thawed in air (*p*-value < 0.01) and polystyrene (*p*-value < 0.05) compared with samples cooled at 1 °C min^−1^, and no significant difference was seen in any other data sets when comparing to the 1 °C min^−1^ cooling condition. Very poor (<2%) viability was seen in samples which were rapidly frozen (cooled at 159 °C min^−1^) therefore the data is not shown here.

### Viable cell number

**Figure 5:**
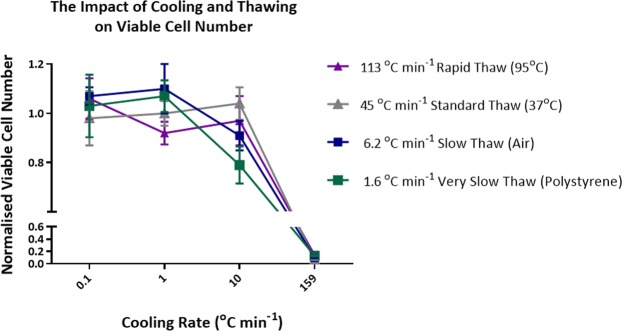
Normalised viable cell numbers achieved at each cool/thaw condition tested. Normalised viable cell number = average (viable cell number/average run control viable cell number). Error bars represent the standard deviation of five replica thaws for the majority of the data shown. Error bars represent the standard deviation of ten replica thaws when cooling at 0.1 °C min^−1^ and thawing in polystyrene and in a 95 °C water bath. Error bars represent the standard deviation of seven replica thaws when cooling at 1 °C min^−1^ and thawing in a 95 °C water bath. The 95 °C water bath data has a dashed line to represent that an inverse trend was observed compared to all other trend lines.

Figure 5 shows the viable cell number immediately post thaw of samples cooled and thawed at different rates, the data is normalised against a standard cryopreservation protocol run on eight different days (Supplementary Materials Fig. S1). For samples thawed in a 37 °C water bath there is no significant difference between the data sets at each of the cooling rates tested. The most noteworthy result was from samples cooled at 10 °C min^−1^ which resulted in significantly worse outcome when thawed in air (*p*-value < 0.05) or polystyrene (*p*-value < 0.01) compared with those cooled at 1 °C min^−1^. Samples thawed in a 95 °C water bath had significantly (*p*-value < 0.01) better post thaw viable cell numbers when cooled at 0.1 °C min^−1^ compared with cooling at 1 °C min^−1^.

### Average and normalized proliferation

**Figure 6:**
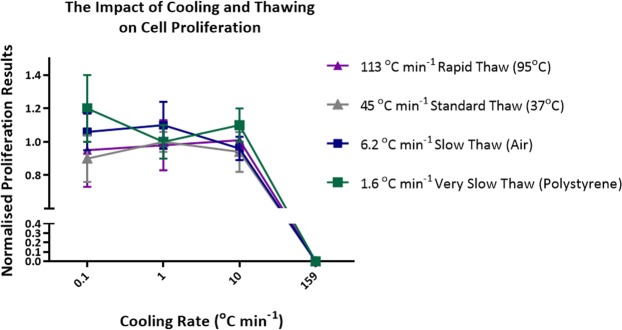
Normalised proliferation results achieved at each freeze/thaw condition tested. Normalised proliferation = average (percentage proliferation/average run control percentage proliferation). Error bars represent the standard deviation of five replica thaws for the majority of the data shown. Error bars represent the standard deviation of ten replica thaws when freezing at 0.1 °C min^−1^ and thawing in a 95 °C water bath. Error bars represent the standard deviation of seven replica thaws when freezing at 1 °C min^−1^ and thawing in a 95 °C water bath.

Figure 6 shows that proliferation four days after thaw was not affected by either cooling rates or warming rates – no significant differences were seen. However, more intra-experimental variation was seen in proliferation studies as highlighted in Fig. S1.

### Phenotype assessment

For all conditions tested in this study (except for those which were plunged into LN_2_ at 159 °C min^−1^, resulting in too few live cells to be successfully processed), it was found that the phenotype of the cells before cryopreservation and after warming matched with >95% CD3 positive cells. Therefore, the detrimental impact on viable cell number observed at particular conditions in this study had no impact on the cell phenotype profile.

### Comparison against literature

**Figure 7:**
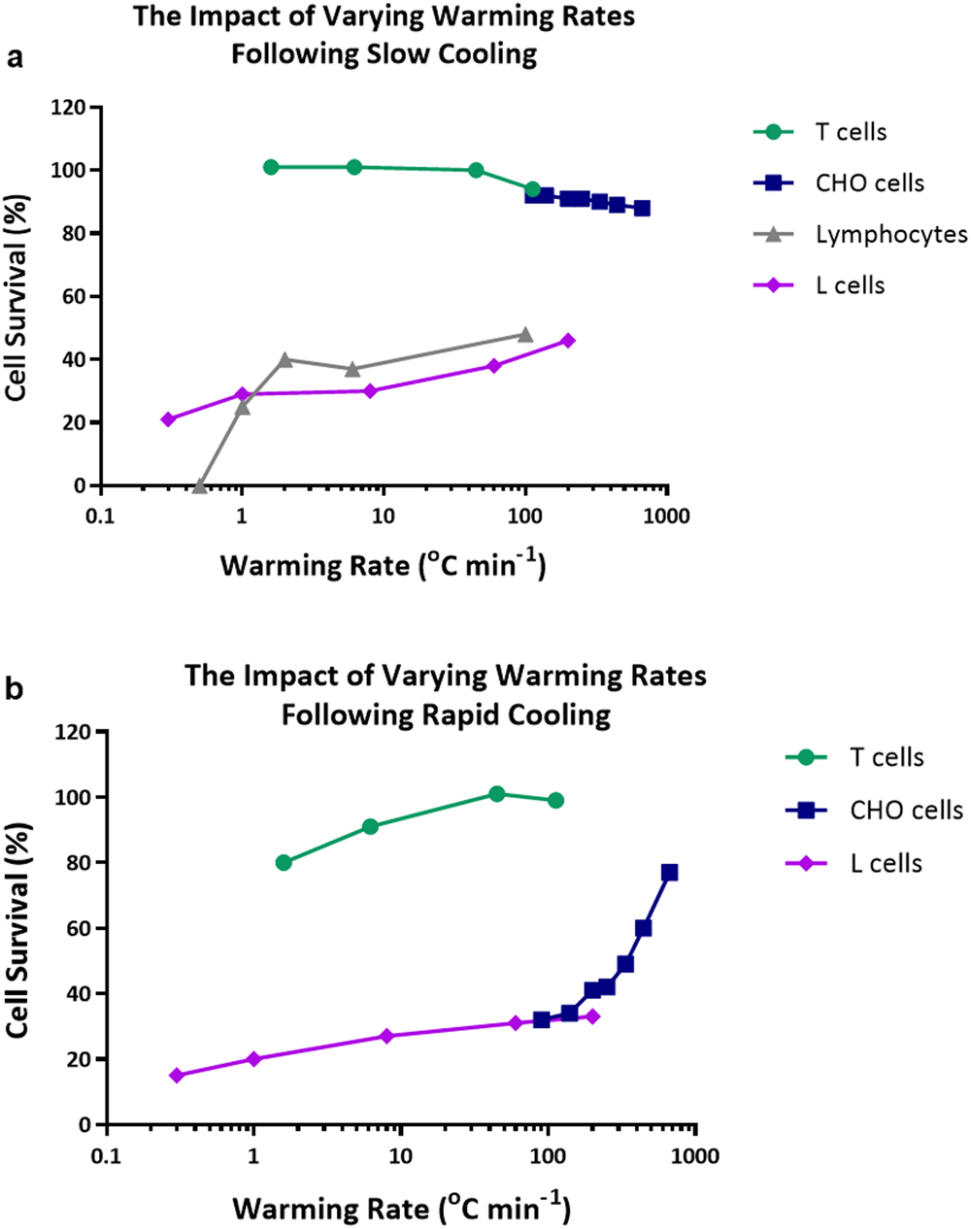
Literature values showing the effect of different rates of warming following either (**a**) Slow cooling; L cells following cooling at 1 °C min^−1^ (♦)^25^, CHO cells following cooling at 1.7 °C min^−1^ (▪)^24^, lymphocytes following cooling at 2.7 °C min^−1^ (▲)^12^ and T cells following cooling at 1 °C min^−1^ (●) (this paper) or (**b**) Rapid cooling; L cells following cooling at 10 °C min^−1^ (♦)^25^, CHO cells following cooling at 100 °C min^−1^ (▪)^24^ and T cells following cooling at 10 °C min^−1^ (●) (this paper).

In Fig. 7 we replot literature values of the effect of warming rate on the viability of somatic mammalian cells following either slow or rapid cooling. At slow rates of cooling (Fig. 7A) there was very little effect of warming rate on survival for T cells redrawn from Fig. 6 and CHO cells, with L cells viability was reduced by 36% when the warming rate was reduced from 200 °C min^−1^ to 1 °C min^−1^, at a slower rate (0.3 °C min^−1^) there was a further 20% reduction in viability. With lymphocytes cooled at an intermediate rate of cooling (2.7 °C min^−1^) viability was reduced by 17% when the warming rate was reduced from 100 °C min^−1^ to 2 °C min^−1^; at slower rates (1 °C min^−1^), there was significant reduction in viability and at a rate of 0.5 °C min^−1^ no viable cells were recovered.

Following rapid cooling rates (Fig. 7B) the viability of T cells and CHO cells was significantly reduced at slow rates of warming. With L cells the influence of warming rate following fast cooling was very similar to that observed following slow cooling.

### Scale-Up

Conventional cryovials used in the study are manufactured from polypropylene, whilst new vials and bags are manufactured from Cyclic Olefin Copolymer (COC) and Ethylene vinyl acetate (EVA), respectively. Values of typical wall thickness and heat conductivity are shown in Table 2. Bags are expected to thaw faster than cryovials as they have a larger surface area to volume ratio and thinner walls aiding thermal conductivity.

**Table 2:** Some physical properties of the wall materials of conventional cryovials (polypropylene), Aseptic Technologies cryovials (COC), CellSeal vials (COC) West Pharma (COC), cryobags (EVA) and aluminium cassettes.

Wall material	Thermal conductivity, (approx.)	Wall Thickness	Relative conductivity^(1)^
Polypropylene	0.15 W m^−1^ K^−1^	1.0 mm	1
Cyclic olefin copolymer (COC)	0.13 W m^−1^ K^−1^	1.0 mm	0.9
Ethylene-vinyl acetate (EVA)	0.25 W m^−1^ K^−1^	0.3 mm (without overwrap)	5.6
Ethylene-vinyl acetate (EVA)	0.25 W m^−1^ K^−1^	0.6 mm (with overwrap)	2.8
Aluminium	200 W m^−1^ K^−1^	variable	Typically >100

The relative conduction rates relative to a polypropylene cryovial have been calculated during thawing, i.e. a value of 2 indicates that heat will conduct twice as fast through the wall, and 0.5 half as fast.

## Discussion

Most texts in cryobiology maintain that rapid thawing is essential for optimum cell recovery^17–21,33–35^. An established view which has become embedded into current thinking in cell biology and cell therapy, the reasons for this could be:

- No systematic studies for 35 years, last systematic study for mammalian somatic cells was published in 1979, by contrast over the same period at least 6 papers were published on warming rates for cryopreserved sperm and at least 5 on warming rates for cryopreserved embryos.
- Assumption that data from sperm and embryos translated to somatic cells.
- Slow thawing – confounding of post thaw DMSO toxicity and slow thawing^36,37^.

In the current study we have shown that this is an over simplification and Table 3 summarises the complex picture of the effects of warming rate on cell viability for a range of cooling rates and cell types. The results show that when T cells are frozen in DMSO at slow rates of cooling there is no effect of warming rate on cell viability, this is consistent with early studies on other mammalian cells^24,25^. The only published literature on lymphocytes^12^ is confused by an intermediate rate of cooling (2.7 °C min^−1^) and the addition of foetal calf serum to the DMSO cryoprotectant. But in this publication little effect of rate of warming is observed in the range 200 °C min^−1^ to 2 °C min^−1^.

**Table 3:** The effects of warming rate on cell viability for a range of cooling rates and cell types.

Cell Type	Cooling Rate	Cryoprotectant	Effect of warming rate	References
Mammalian cells in suspension (T cells etc)	“Slow” eg 1	DMSO	Little effect of warming rate on viability.	Fig. 8^24,25,37^
Mammalian cells in suspension (T cells etc)	“Fast” >10	DMSO	Rapid thawing beneficial.	^44^
Mammalian sperm	“Fast”	Glycerol	Rapid thawing beneficial.	^22^
Mammalian embryos	“Slow”	DMSO and Glycerol Studied	Slow thawing beneficial.	^45^
Mammalian biopsies, islets encapsulated cells,	“Slow”	DMSO	Rapid thawing beneficial.	^46– 48^
Bacteria	“Fast”	Glycerol	“fast freezing followed by fast thawing resulted in greatest survival”.	^49^
Erythrocytes	“Slow”	Glycerol	Following slow rates of cooling, high survival at slow rates of warming.	^50^
Erythrocytes – at very high cell density (75% haematocrit)	“Slow”	Glycerol	“dependent on warming rate at cooling rates <100 °C/min and on cooling rate at higher cooling rates”.	^51^
Rapid cooling in the presence of inhibitors	“Rapid”	None	“Lowering the thawing rate … significantly reduced RBC recovery”.	^52, 53^
Vitrification	“Rapid”	DMSO	“Rapid cooling and thawing required to prevent any ice in system – unlike conventional freezing studied here”.	^54^

Non-linear warming rates are observed during thawing of cryovials (Fig. 2) because the ice fraction is not linear with temperature (between nucleation and −10 °C, 75% of ice is formed, by contrast between −30 °C and −40 °C, 3.5% of ice forms (Fig. 2)^38,39^. Thawing was commenced at −100 °C to ensure consistency between the samples. It has been shown that cycling samples from −196 to −102 °C has no effect on cell viability and function, and that a short term hold on warming at dry ice temperatures does not impact cell recovery^14,40^. As such, in practice warming directly from a storage temperature without any hold step would not be expected to vary the results, nor would having different start temperatures between multiple samples below −100 °C, a situation which may occur if samples are stored in a vapour phase tank where thermal stratification can occur. Many therapies are stored separately from the site of thawing, and so must be transported in a cooled process.

Cryomicroscopy revealed that the morphology of the crystals formed during cooling is determined by the rate of cooling, with large crystals being observed at very slow rates of cooling (0.1 °C min^−1^) and finer dendritic structures following a cooling rate of 10 °C min^−1^. Following rates of cooling of 0.1 °C min^−1^ and 1 °C min^−1^ there were no discernible changes in ice structure during warming irrespective of the warming rate, (see Fig. 3 and Supplementary Videos). DSC also revealed recrystallization but at higher cooling rates than 10 °C min^−1^, probably due to different heat transfer in DSC device/sample (all water crystallises at 10 °C min^−1^) than in cryomicroscopy/sample (some water crystallises during slow warming if cooling at 10 °C min^−1^ or faster). Following a higher cooling rate of 10 °C min^−1^ significant recrystallization was observed at slow rates of warming but not rapid rates (see Fig. 3 and Supplementary Videos). At slow rates of cooling “equilibrium” crystallization will be expected (i.e., all the freezable water has crystallized as per the system’s phase diagram) and as the rate of cooling increases “non-equilibrium” crystallization will occur which will lead to the potential of recrystallization on warming. In the case of non-equilibrium crystallization, the system cools faster (and so solution gets more viscous) more rapidly than water molecules can diffuse onto the ice crystal. This results in crystallization resuming as the sample moves through the thawing regime (provided thawing rates are low enough to allow this re-crystallization to occur before the system reaches its melting point). In the event both cooling and warming rates are high enough, the crystallization process will not run to completion. It is important to note that this process is highly dependent on medium viscosity. On cooling as the ice fraction increases, cells experience a hypertonic environment and dehydrate accordingly. As these ice crystals melt on warming the cell returns to a lower osmotic environment and remain viable. However, on warming following non-equilibrium cooling ice crystallization resumes exposing the cell to an increase in concentration before melting occurs^41^. The extent of recrystallization following a rapid rate of cooling will be determined by the warming rate; they will be minimised at rapid rates and increase as the rate of warming decreases. We have previously examined this phenomenon with the cryoprotectant glycerol^42^ and DMSO^43^. Our DSC data here shows that the ice fraction formed in the presence of DMSO as a cryoprotectant is sensitive to cooling rate, but to a lower extent than seen previously with glycerol^42,43^ (Table 1 column ΔHc, and Fig. 4 which shows two endothermic peaks representing glass transition). This may be due to viscosity effects, the viscosity of glycerol in the residual unfrozen fraction increases dramatically during freezing^42^ whilst the measured viscosity of DMSO in the unfrozen fraction during freezing is less dramatic^43^. At higher viscosity the rate of growth of ice during cooling is limited.

Varying viability results were obtained with the two assay methods (Supplementary Fig. S2). With Trypan blue very little differential between all treatments was observed, whereas larger differences were observed with the live/dead aqua stain and the most significant result was a reduction in viable cells following a combination of rapid cooling/slow thawing. The same pattern was observed when the viable cell number was determined (Fig. 5). Cell proliferation studies (Fig. 6) show that for cells which survived the freeze thaw protocol, proliferation rate was unaffected. One novel observation in these data sets are the very high viability, total viable cell numbers and cell proliferation observed following very slow rates of cooling (0.1 °C min^−1^).

**Figure 8:**
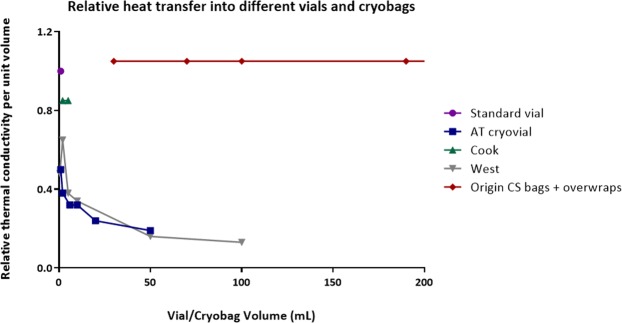
Thawing rates normalised against standard 1 mL cryovials for a range of hermetically sealable cryovials and for cryobags (2D and 3D) thawed in different orientations. Calculations made for different cryocontainers filled to the manufacturers recommended fill volumes considering container thickness, material, and surface area to volume ratios. This takes into account how much energy reaches each ml of frozen vial content, as well as surface area (from Table 2).

Moving forward, demands of clinical cell therapy require that hermetically sealed vials and bags as well as larger sample volumes in different formats (bags or vials) are to be used. All these factors have implications on the rate of thawing available within samples. Conventional cryovials used in the study are manufactured from polypropylene, whilst new vials and bags are manufactured from COC and EVA respectively. Values of typical wall thickness and heat conductivity are shown in Table 2. As can be seen from Fig. 8, while larger volumes take longer to thaw, this is offset by the thinner bags in which they are usually contained, and the flat nature of bags maximizes surface area to volume ratios. While this work found that rapid or slow thawing have equal results after slow cooling, practically faster or slower thawing may be preferable depending on manufacturing protocols.

## Conclusion

This study demonstrates that with suitable control of the cooling rates, the thawing of cell therapy products is more robust in the face of practical clinical differences at bedside delivery. This will result in reduced variability at the clinic – the least controlled step in cold chain delivery – enabling more effective and predictable clinical outcomes. Cell therapies can take advantage of this data to cooling slowly to protect cells upon thaw, and this work provides the first explanation coupling imagery, calorimetry, and laboratory data to explain the inter-relationship between cooling and warming rates for these therapies.

## Supplementary information

Supplementary Figures
