# Supplementary material for: The Impact of Varying Cooling and Thawing Rates on the Quality of Cryopreserved Human Peripheral Blood T Cells
Source: Sci Rep. 2019 Mar 4;9:3417. doi: 10.1038/s41598-019-39957-x (PMC6399228; doi:10.1038/s41598-019-39957-x)
Supplement: Supplementary file 1 — Supplementary Figures [file 41598_2019_39957_MOESM1_ESM.docx]

**The Impact of Varying Cooling and Thawing Rates on the Quality of Cryopreserved Human** **Peripheral Blood T Cells**

**Authors** Jasmin Baboo^1^, Peter Kilbride^2^, Mike Delahaye^1^, Stuart Milne^2^, Fernanda Fonseca^3^, Magdalena Blanco^1^, Julie Meneghel^2^, Alex Nancekievill^2^, Nick Gaddum^1^, John Morris^2^

**Authors Affiliation**

1 – Cell and Gene Therapy Catapult, 12th Floor Tower Wing, Guy's Hospital, Great Maze Pond, London SE1 9RT, UK

+44 20 3728 9500

2 – Asymptote, General Electric Healthcare, Sovereign House, Histon, Cambridge, CB24 9BZ, UK

+44 330 660 0320

3 – UMR GMPA, AgroParisTech, INRA, Université Paris Saclay, 78850 Thiverval-Grignon, France

+33 1 30 81 59 40

**Contact**

John Morris; [GJohn.Morris@ge.com](mailto:GJohn.Morris@ge.com); +44 330 660 0320

**Supplementary Materials**

**Figure S1.** Box and whisker plots displaying the distribution in viable cell numbers recovered and percentage proliferation over eight experimental runs for vials thawed in a 37^o^C water bath which had been cooled at 1^o^C min^-1^ to -100^o^C. The whiskers represent the maximum and minimum values obtained in the dataset.

Considering reproducibility of results, Figure S1 shows that experiments run on eight different days resulted in a standard deviation of 5.8% in average viable cell number. This cryopreservation protocol of samples cooled at 1°C min^-1^ to -100°C and thawed in a 37°C water bath has previously been used in this laboratory and was used as a standard control to normalize against.

**Figure S2.** Average cell viability measured at each freeze/thaw condition tested using trypan blue staining (a) and live/dead aqua staining (b). Error bars represent the standard deviation of five replica thaws for the majority of the data shown. Error bars represent the standard deviation of ten replica thaws when freezing at 0.1^o^C min^-1^ and thawing in a 95^o^C water bath and in polystyrene (for trypan blue staining data only). Error bars represent the standard deviation of seven replica thaws when freezing at 1^o^C min^-1^ and thawing in a 95^o^C water bath (for trypan blue staining data only).
